# SIRT7 Is a Prognostic Biomarker Associated With Immune Infiltration in Luminal Breast Cancer

**DOI:** 10.3389/fonc.2020.00621

**Published:** 2020-05-12

**Authors:** Qin Huo, Zhenwei Li, Lixin Cheng, Fan Yang, Ni Xie

**Affiliations:** ^1^Biobank, Shenzhen Second People's Hospital, First Affiliated Hospital of Shenzhen University, Shenzhen, China; ^2^Institute of Translational Medicine, Shenzhen Second People's Hospital, First Affiliated Hospital of Shenzhen University, Shenzhen, China; ^3^Shenzhen People's Hospital, First Affiliated Hospital of Southern University of Science and Technology, Shenzhen, China

**Keywords:** sirtuin 7 (SIRT7), gene expression, tumor-infiltrating, prognosis, breast cancer

## Abstract

**Background:** Sirtuin 7 (SIRT7), a protein-coding gene whose abnormal expression and function are associated with carcinogenesis. However, the prognosis of SIRT7 in different breast cancer subtypes and its correlation with tumor-infiltrating lymphocytes remain unclear.

**Methods:** The expression and survival data of SIRT7 in patients with breast cancer were analyzed using Tumor Immune Estimation Resource (TIMER), Gene Expression Profiling Interaction Analysis (GEPIA), The Human Protein Atlas (HPA), UALCAN, Breast Cancer Gene-Expression Miner (BC-GenExMiner), and Kaplan-Meier plotter databases. Also, the expression correlations between SIRT7 and immune infiltration gene markers were analyzed using TIMER and further verified the results using immunohistochemistry.

**Results:** SIRT7 exhibited higher expression levels in breast cancer tissues than the adjacent normal tissues. SIRT7 expression was significantly correlated with sample type, subclass, cancer stage, menopause status, age, nodal status, estrogen receptor (ER), progesterone receptor (PR), and triple-negative status. High SIRT7 expression was associated with poor prognosis in breast cancer-luminal A [overall survival (OS): hazard ratio (HR) = 1.54, *p* = 1.70e-02; distant metastasis-free survival (DMFS): HR = 1.56, *p* = 2.60e-03]. Moreover, the expression of SIRT7 was positively correlated with the expression of IRF5 (M1 macrophages marker, *r* = 0.165, *p* = 1.13e-04) and PD1 (T cell exhaustion marker, *r* = 0.134, *p* = 1.74e-03). These results suggested that the expression of SIRT7 was related to M1 macrophages and T cell exhaustion infiltration in breast cancer-luminal.

**Conclusions:** These findings demonstrate that the high expression of SIRT7 indicates poor prognosis in breast cancer as well as increased immune infiltration levels of M1 macrophages and T cell exhaustion in breast cancer-luminal. Thus, SIRT7 may serve as a candidate prognostic biomarker for determining prognosis associated with immune infiltration in breast cancer-luminal.

## Introduction

Breast cancer is the most common malignant disease affecting women worldwide (1). Most breast cancer-related deaths are caused by metastases (2). Based on gene expression profiles, breast cancer is classified into three main subtypes: luminal (luminal A and luminal B), human epidermal growth factor receptor (HER2)-positive, and triple-negative breast cancer (TNBC) (3). A large number of patients experienced relapse due to organ metastases, especially those with the worst TNBC prognosis (4). In addition, a study reported that patients with distant metastatic breast cancer faced a 5-year survival rate of less than 25% (5). Currently, surgery, radiotherapy, chemotherapy, endocrine therapy, and other combined therapies are commonly used in the clinic with certain effects (6). However, these conventional therapies work only at the early stages but not that effective for patients at advanced stages or with distant metastasis.

Over the past decade, several studies demonstrated the role of adaptive immune response in regulating cancer growth. Recently, a study published in the *Cancer Cell* identified key changes in immune cells in cancerous tumors that may help predict the prognosis of cancer patients (7). The results may help researchers develop new technologies to diagnose and predict the survival status of breast cancer patients and to study the behavioral characteristics of other common cancer lesions. The absolute number of tumor-infiltrating lymphocytes (TILs) is essential to confer potential protective immunity against tumor (8) and may be an independent prognostic factor for some tumors (9). Murray et al. (10) reported that the patients with high TILs in tumor tissue had a better prognosis than patients with low TILs. Subsequently, several studies have demonstrated this phenomenon in diverse tumor types, including breast cancer (11), colorectal cancer (12), and renal cell cancer (13). Many authors attempted to describe the immune response to breast cancer to evaluate its role and efficacy as a prognostic marker of cancer (14). The prognosis of breast cancer is related to not only the biological characteristics but also the tumor microenvironment (15). CD8^+^ lymphocyte is a crucial member of the tumor microenvironment that mediates tumor-specific immune responses. In breast cancer patients, high infiltration of CD8^+^ lymphocytes predicts a significantly higher response to chemotherapy compared to low infiltration (16). Previous studies on the prognostic value of invasive immune cells in breast cancer reported that numerous invasive CD8^+^ cytotoxic lymphocytes in tumor tissues were significantly associated with the survival and prognosis of patients (17). Macchetti et al. (18) performed a flow cytometric analysis of TILs in 23 patients with T1–T2 breast cancer. They found that the average percentage of tumor-infiltrating CD4^+^ T cells increased, rather than CD8^+^ T cells, and that this infiltration was associated with poor patient prognosis. A recent study has demonstrated that stromal TILs can predict the disease-free survival in patients with HER2^+^ breast cancer to some extent (19). Therefore, there is an urgent need to identify novel immune-related therapeutic targets in breast cancer.

Sirtuins (SIRTs) belong to the nicotinamide adenine dinucleotide (NAD^+^)-dependent histone deacetylases (HDACs) Class III family, which are highly conserved between bacteria and human (20). SIRTs are implemented in pathways of DNA repair, inflammation, aging, and cell survival through substrate deacetylation (21). SIRT7, a key member of the SIRTs family, is involved in several physiological processes, including rRNA transcription and modification, cellular metabolism, cellular stress, and DNA damage repair. Tang et al. (4) reported that SIRT7 was significantly downregulated in human and mouse breast cancer with lung metastasis. Additional studies have shown that resveratrol-dependent activation of SIRT7 deacetylase activity can antagonize transforming growth factor (TGF)-β signaling, inhibit epithelial-mesenchymal transition, and ultimately inhibit lung metastasis of breast cancer, thereby improving survival rates. It has been reported that certain genes, such as LAYN and BRD4, have multiple functions in TILs (22–24). However, the potential functions of SIRT7 in tumor progression and tumor immune response remain unclear.

In this study, we comprehensively investigated the expression pattern of SIRT7 and its association with the prognosis of breast cancer patients using several bioinformatics web servers including Tumor Immune Estimation Resource (TIMER), Gene Expression Profiling Interaction Analysis (GEPIA), The Human Protein Atlas (HPA), UALCAN, Breast Cancer Gene-Expression Miner (BC-GenExMiner), and Kaplan-Meier plotter. We also evaluated the relationship between SIRT7 expression levels and different clinical pathological parameters of breast cancer, such as sample type, patient's age, cancer stage, and breast cancer subclass. Next, we compared the expression levels of SIRT7 association with prognosis in different breast cancer subtypes. Moreover, we investigated the correlation between SIRT7 expression and immune infiltration levels in breast cancer by TIMER database and immunohistochemistry.

## Methods

### Tumor Immune Estimation Resource Database Analysis

TIMER (https://cistrome.shinyapps.io/timer/) is a comprehensive resource for systematic analysis of immune infiltrates across diverse cancer types (25). It includes 10,897 samples from 32 cancer types to estimate the role of immune infiltration. The infiltration of immune cells in tumor tissues can be detected and quantified from the RNA-seq expression profile data, thereby determining the relationship between tumor and immune cells. Moreover, the database can accurately quantify the purity of tumors and the immune infiltration levels and assess the correlation between infiltration and clinical prognosis. We analyzed the expression of SIRT7 in different cancer types by gene module and the correlation between SIRT7 expression and immune infiltration, including B cells, CD4^+^ T and CD8^+^ T cells, neutrophils, macrophages, and dendritic cells. Different cancer types (tumor/normal) were plotted on the x-axis and SIRT7 expression on the y-axis. The levels of gene expression were represented by log_2_ RSEM.

Also, we explored the correlation between SIRT7 expression and gene markers of immune cells [CD8^+^ T cells, T cells, B cells, monocytes, tumor-associated macrophages (TAMs), M1 and M2 macrophages, neutrophils, natural killer cells, and dendritic cells] through relevant modules. Relevant modules generated scatter maps of expression between a pair of user-defined genes in a given cancer type, as well as the statistical significance of Spearman's correlation and estimation.

### Gene Expression Profiling Interaction Analysis

GEPIA (http://gepia.cancer-pku.cn/) is an interactive web application based on the gene expression analysis of 9,736 tumors and 8,587 healthy tissue samples from The Cancer Genome Atlas (TCGA) and The Genotype-Tissue Expression (GTEx) databases. The analysis results included ~20,000 coding genes, ~25,000 non-coding genes, ~14,000 pseudogenes, and ~400 T-cell receptor fragments (26). In the study, we used the GEPIA database to analyze the expression levels of SIRT7 in breast cancer tissues and normal tissues by the “Expression DIY” tab.

### Human Protein Atlas Analysis

HPA (https://www.Proteinatlas.org/) makes use of antibody method for immunostaining on tissues and cell lines as well as for differential expression analysis of proteins in normal and tumor tissues (27). In this study, we checked the expression of SIRT7 in the protein expression module of the HPA database and analyzed the immunohistochemical results of SIRT7 in tumor tissues and normal tissues (Antibody: HPA053669).

### UALCAN Analysis

UALCAN (http://ualcan.path.uab.edu/) is an effective online analysis and mining website for an in-depth analysis of gene expression data using TCGA levels 3 RNA-seq and clinical data from 31 cancer types (28). It allows the relative expression of genes between tumors and normal samples, as well as in different tumor subgroups based on sample type, individual tumor stage, major subclasses, and other clinical pathological features. We entered the target gene SIRT7 on the home page of the website, selected breast invasive carcinoma, and obtained differential expression of the target gene in breast cancer tissues and normal tissues. This study will analyze the differential expression of SIRT7 from various angles such as sample type (normal/primary tumor), breast cancer subclass (luminal, HER2^+^, and triple-negative), cancer stage (stages 1, 2, 3, and 4), and menopause status (premenopause, perimenopause, and postmenopause).

### BC-GenExMiner 4.4 Analysis

BC-GenExMiner (http://bcgenex.centregauducheau.fr/), a statistical mining tool of published annotated breast cancer transcriptomic data [DNA microarrays (*n* = 10,001) and RNA-seq (*n* = 4,712)] (29, 30). The prognostic module measures the prognostic effect of candidate genes in breast cancer and identifies potential prognostic markers. The correlation module calculates the correlation between genes located in the same chromosomal region or related coexpression gene clusters (31). First, we logged in to the BC-GenExMiner home page, selected RNA-seq TCGA for gene expression data, and entered the gene “SIRT7.” We then analyzed SIRT7 expression levels based on various classified parameters such as age (≤51 and >51); nodal status (N^+^/N^−^); estrogen receptor (ER), progesterone receptor (PR), and HER2 status (ER^+^/ER^−^, PR^+^/PR^−^, HER2^+^/HER2^−^); molecular subtypes; and triple-negative status (TNBC vs. Not TNBC).

### Kaplan-Meier Plotter Database Analysis

The Kaplan-Meier database (http://kmplot.com/analysis/) could assess the effects of 54,675 genes on survival using 10,461 cancer tissue samples, which included 5,143 breast cancer, 1,816 ovarian cancer, 2,437 lung cancer, and 1,065 gastric cancer samples (32). We compared the expression levels of SIRT7 in different breast cancer subtypes (basal, luminal A, luminal B, and HER2^+^). Overall survival (OS) and distant metastasis-free survival (DMFS) were calculated using the Kaplan-Meier method, and the result was shown in **Figure 4**. Hazard ratio (HR) for 95% confidence interval and *p* < 0.05 were considered statistically significant.

### Immunohistochemistry

This study was performed on archived tissues from 10 diagnosed cases of breast cancer-luminal. These samples were obtained from Shenzhen Second People's Hospital. This study was approved by the Ethics Committee of Shenzhen Second People's Hospital in accordance with the principles of the Declaration of Helsinki. To validate the relationship between SIRT7 expression and tumor-infiltrating immune cells, we performed immunohistochemistry to assess SIRT7, IRF5, and PD1. First, the collected tissue samples were fixed and embedded in paraffin to make paraffin tissue sections and placed on slides. The slides were deparaffinized and rehydrated through graded alcohols and were then subjected to an antigen retrieval procedure (10 mM sodium citrate, 0.05 % Tween-20, pH 6.0 for 25 min). Endogenous peroxidase was blocked with 3% H_2_O_2_ (freshly made) for 10 min at room temperature. Then the tissues were incubated by primary antibodies and incubated at 4°C overnight and incubated by secondary antibodies. The concentration of the three antibodies was optimized; the following primary antibodies were used: rabbit anti-SIRT7 (1:100, Affinity Biosciences), rabbit anti-IRF (1:100, Affinity Biosciences), and rabbit anti-PD1 (1:200, Affinity Biosciences). MXB was used to detect secondary antibodies. The color reaction was then carried out using 3′-diaminobenzidine (DAB). The expression density of SIRT7, IRF5, and PD1 in breast cancer tissue was quantitated by scoring staining intensity, including negative (–) and weak (+) staining, moderate (++) and strong (+ + +) staining, respectively (33).

### Statistical Analysis

The differences in OS and DMFS between high risk and low risk of SIRT7 were analyzed using Kaplan-Meier method. The results of Kaplan-Meier plots and GEPIA were presented with HRs and *p*-values from a log-rank test. Spearman correlation coefficient was used to measure the expression correlation among genes. A value of *p* < 0.05 was considered statistically significant.

## Results

### High Expression of Sirtuin 7 in Breast Cancer

**Figure 1:**
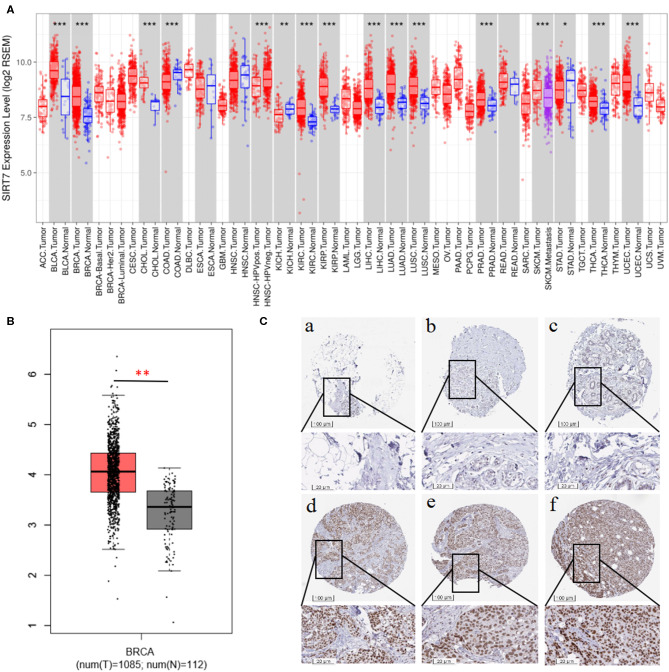
**(A)** Human sirtuin (SIRT)7 expression levels in different tumor types from The Cancer Genome Atlas (TCGA) database were determined using Tumor Immune Estimation Resource (TIMER). Compared with normal tissues, SIRT7 was upregulated in breast, bladder urothelial carcinoma (BLCA), cholangiocarcinoma (CHOL), kidney renal clear cell carcinoma (KIRC), kidney renal papillary cell carcinoma (KIRP), liver hepatocellular carcinoma (LIHC), lung adenocarcinoma (LUAD), lung squamous cell carcinoma (LUSC), prostate adenocarcinoma (PRAD), thyroid carcinoma (THCA), and uterine corpus endometrial carcinoma (UCEC) and downregulated in colon adenocarcinoma (COAD) and kidney chromophobe (KICH) cancers. **(B)** Gene Expression Profiling Interaction Analysis (GEPIA) for the expression of SIRT7 in tumor tissues and normal tissues. TCGA data revealed that SIRT7 mRNA was significantly higher in breast cancer tissues (1,085 cases) than that in normal tissues (112 cases). **(C)** SIRT7 expression in breast cancer tissues and normal tissues from the Human Protein Atlas (HPA) database. Immunohistochemical staining revealed that SIRT7 exhibited low expression in normal tissue samples (a–c) and high expression in tumor tissues (d–f). *p*-value significant codes: 0 ≤ *** < 0.001 ≤ ** < 0.01 ≤ * < 0.05 ≤. < 0.1.

We examined the difference in SIRT7 expression between tumor and adjacent normal tissues by using RNA-seq data from multiple malignancies in TCGA. The results were shown in Figure 1A. From the TIMER database, we found that SIRT7 was upregulated in breast cancer, cholangiocarcinoma, kidney renal clear cell carcinoma, kidney renal papillary cell carcinoma, liver hepatocellular carcinoma, lung adenocarcinoma, lung squamous cell carcinoma, prostate adenocarcinoma, thyroid carcinoma, and uterine corpus endometrial carcinoma, while it has lower expression levels in colon adenocarcinoma and kidney chromophobe cancers compared with adjacent healthy tissues.

To validate the results in breast cancer, GEPIA was used to analyze 1,197 samples from the TCGA database. As shown in Figure 1B, TCGA data revealed that SIRT7 mRNA was significantly higher in breast cancer tissues (1,085 cases) than that in normal tissues (112 cases) (*p* < 0.01), which is consistent with the TIMER database. In addition, we used the antibody HPA053669 of SIRT7 to analyze the immunohistochemical results of normal and tumor tissues using HPA and found that the protein expression and antibody staining level of three cases of breast cancer were moderate (Figure 1C). This further confirmed that the expression of SIRT7 in tumor tissues was significantly higher than that in normal tissues.

### Relationship Between Sirtuin 7 Expression and Clinical Pathological Parameters of Patients With Breast Cancer

**Figure 2:**
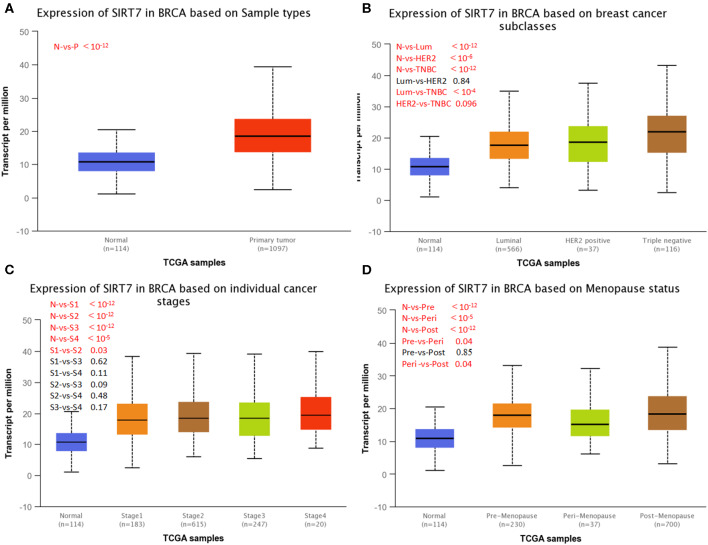
UALCAN analysis for the correlation between sirtuin (SIRT)7 mRNA expression level and clinicopathological parameters of breast cancer. **(A)** Sample type (normal/primary tumor). **(B)** Breast cancer subclass (luminal, HER2^+^, and triple negative). **(C)** Cancer stage (stages 1, 2, 3, and 4). **(D)** Menopause status (premenopause, perimenopause, and postmenopause). N, normal; P, primary tumor; Lum, luminal; HER2, HER2^+^; TNBC, triple negative; S1, stage 1; S2, stage 2; S3, stage 3; S4, stage 4; Pre, premenopause; Peri, perimenopause; Post, postmenopause; BRCA, breast cancer.

UALCAN is used to study gene expression levels based on TCGA data and clinical patient data. It is used not only to compare primary tumors and healthy tissue samples but also to compare clinical pathological parameters of patients based on pathological staging, tumor grade, and other clinical pathology features. We next investigated SIRT7 expression on the basis of patients' different clinical pathological parameters, such as sample type (normal/primary tumor), breast cancer subclass (luminal, HER2^+^, and triple-negative), cancer stage (stages 1, 2, 3, and 4), and menopause status (premenopause, perimenopause, and postmenopause) using UALCAN database. As shown in Figure 2A, the expression of SIRT7 in breast cancer samples was significantly higher than that in normal breast tissues (*p* < 10^−12^). An analysis of subclass showed that SIRT7 was higher expressed in different subclasses than that in normal breast tissues (normal vs. breast cancer-luminal, p < 10^−12^; normal vs. HER2-positive breast cancer, *p* < 10^−6^; normal vs. TNBC, *p* < 10^−12^). Compared with subtypes of luminal and HER2, SIRT7 was higher expressed in the three negative subtypes (luminal vs. TNBC, *p* < 10^−4^; HER2 vs. TNBC, *p* = 0.096) (Figure 2B). For cancer stages, the higher expression of SIRT7 in late-stage cancers compared to early stages suggests a possible role of SIRT7 in cancer progression and invasion (Figure 2C). Besides, as shown in Figure 2D, the expression of SIRT7 in postmenopause was higher than that in perimenopause (perimenopause vs. postmenopause, *p* = 0.04). SIRT7 expression in perimenopause was lower than that in premenopause (premenopause vs. perimenopause, *p* = 0.04).

**Figure 3:**
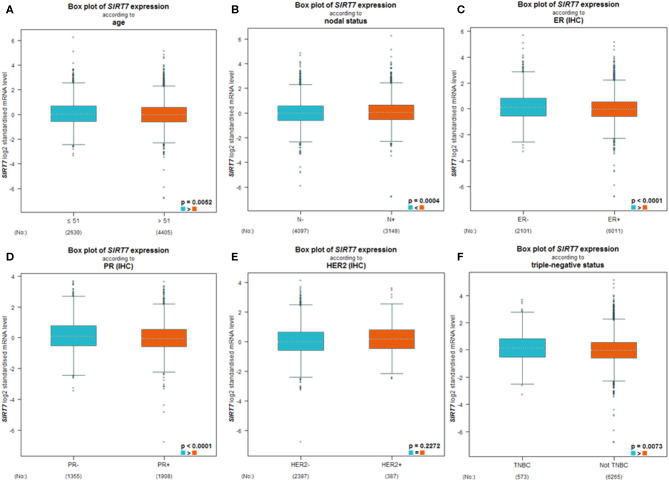
Differential expression levels of sirtuin (SIRT)7 mRNA in patients with breast cancer were performed by bc-GenExMiner v4.4 based on different types of classified parameters: **(A)** age (≤ 51 and >51 years), **(B)** nodal status (N^+^ vs. N^−^), **(C–E)** receptor status (ER^+^ vs. ER^−^, PR^+^ vs. PR^−^, HER2^+^ vs. HER2^−^), **(F)** triple-negative status (TNBC vs. Not TNBC).

BC-GenExMiner 4.4 was used to explore the correlation between SIRT7 mRNA levels and the six common risk factors, namely, age, nodal status, ER status, PR status, HER2 receptor status, and triple-negative status (Table 1). Regarding age, SIRT7 mRNA expression was significantly higher in ≤51 years group than that in >51 years group (*p* = 5.20e-03; Figure 3A). The results showed that there were remarkably different expression levels of SIRT7 mRNA in nodal status (N+ > N–, *p* = 0.0004; Figure 3B), ER status (ER– > ER+, *p* < 0.0001; Figure 3C), PR status (PR– > PR+, *p* < 0.0001; Figure 3D), triple-negative status (TNBC > not TNBC, *p* = 0.0073; Figure 3F), respectively. However, no significant expression difference of SIRT7 mRNA was found in HER2 receptor status (Figure 3E). These results suggest that SIRT7 expression may serve as a potential diagnostic indicator in breast cancer.

**Table 1:** Relationship between mRNA expression of SIRT7 and clinicopathological parameters of breast cancer.

**Variables**	**SIRT7**	
	***N***	***P*-value**
**Age**		
≤51	2,630	5.20e-03
>51	4,405	
**Nodal status**		
−	4,097	4.00e-04
+	3,149	
**Estrogen receptor status (ER) (IHC)**		
−	2,101	<1.00e-04
+	6,011	
**Progesterone receptor status (PR) (IHC)**		
−	1,355	<1.00e-04
+	1,908	
**HER2 receptor status (HER2) (IHC)**		
−	2,387	2.27e-01
+	387	
**Triple-negative status**		
TNBC	573	7.30e-03
Not TNBC	6,265	

### Decreased mRNA Expression of Sirtuin 7 Was Associated With Better Overall Survival and Distant Metastasis-Free Survival in Patients With Breast Cancer-Luminal A

**Figure 4:**
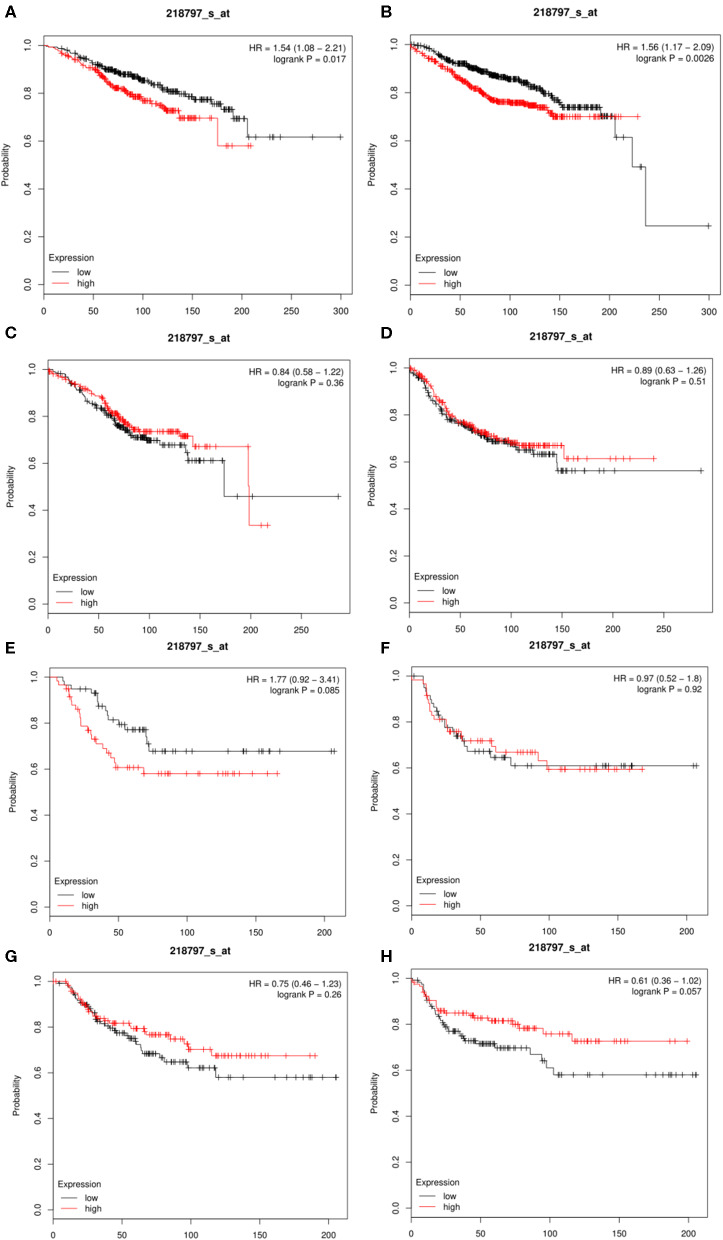
Kaplan-Meier survival curves comparing the high and low expressions of SIRT7 in different breast cancer subtypes (luminal A, luminal B, HER2^+^, and basal). **(A,B)** Survival curves of overall survival (OS) and distant metastasis-free survival (DMFS) in breast cancer-luminal A (*n* = 2,504). **(C,D)** Survival curves of OS and DMFS in breast cancer-luminal B (*n* = 1,425). **(E,F)** Survival curves of OS and DMFS in breast cancer-HER2^+^ (*n* = 335). OS and DMFS survival curves of breast cancer (*n* = 1,402, *n* = 1,747). **(G,H)** Survival curves of OS and DMFS in breast cancer-basal (*n* = 879).

The results showed that the expression of SIRT7 mRNA in the breast cancer group was higher than that in the normal group. Therefore, the relationship between SIRT7 transcription level and tumor prognosis warranted exploration. In this study, to determine whether SIRT7 can be used as a prognostic biomarker, we compared the expression levels of SIRT7 association with prognosis in different breast cancer subtypes (luminal A, luminal B, HER2^+^, and basal) using Kaplan-Meier survival curves. Notably, SIRT7 expression significantly affected the prognosis of different subtypes. In breast cancer-luminal A, high SIRT7 expression was associated with poor prognosis (OS: HR = 1.54, *p* = 1.70e-02; DMFS: HR = 1.56, *p* = 2.60e-03) (Figures 4A,B). In contrast, no significant correlation was observed between the expression of SIRT7 and the prognosis in breast cancer-luminal B, breast cancer-HER2^+^, and basal (Figures 4C–H). These findings indicate that SIRT7 expression may be a prognostic indicator of mortality risk in patients with breast cancer-luminal A.

### Sirtuin 7 Expression Is Correlated With the Immune Infiltration Levels in Breast Cancer

TILs are independent predictors of sentinel lymph node status and survival in patients with cancer (34, 35). Studies showed that the levels of stroma and immune cells in tumor tissue may be related to the clinical features of the disease. Moreover, the infiltration of tumor-associated normal cells may affect the genomic analysis of clinical tumor samples (36). Therefore, we investigated whether the expression of SIRT7 was correlated with the immune infiltration levels in breast cancer using TIMER. Most of the homologous data in TIMER are from TCGA (25), which can be used to detect and quantify the immune infiltration levels in tumor tissues from the expression profile data of RNA-seq.

We used the TIMER database to study the correlation between SIRT7 and various immunocytochemical markers of immune cells (CD8^+^ T cells, T cells, B cells, monocytes, TAMs, M1 and M2 macrophages, neutrophils, natural killer cells, and dendritic cells) in different breast cancer subtypes (luminal, basal, and HER2^+^). Tumor purity is an important factor affecting immune infiltration analysis of clinical tumor samples (36). Therefore, we showed that SIRT7 expression is significantly related to tumor purity in different breast cancer subtypes. The results showed that the expression levels of SIRT7 were correlated with most markers of different immune cells in breast cancer subtypes (breast cancer-luminal, *n* = 611; breast cancer-basal, *n* = 139; breast cancer-HER2^+^, *n* = 67). Particularly, the expression levels of SIRT7 were significantly positively correlated with seven gene markers in breast cancer-luminal (Table 2), such as CD19 (*r* = 0.105, *p* = 1.46e-02), IRF5 (*r* = 0.165, *p* = 1.13e-04), KIR2DL1 (*r* = 0.106, *p* = 1.31e-02), T-bet (*r* = 0.103, *p* = 1.00e-02), FOXP3 (*r* = 0.12, *p* = 5.06e-03), PD1 (*r* = 0.134, *p* =1.74e-03), and GZMB (*r* = 0.108, *p* = 1.14e-02). However, the expression levels of SIRT7 were only significantly negatively correlated with two gene markers in breast cancer-luminal, such as VSIG4 (*r* = −0.108, *p* = 1.16e-02) and BDCA-1 (*r* = −0.114, *p* = 7.69e-03). These findings implicate that SIRT7 plays a specific role in immune cell infiltration, such as B cells, M1 macrophages, M2 macrophages, natural killer cells, dendritic cells, Th1 marker neutrophils, regulatory T cells, and T cell exhaustion, especially M1 macrophages and T cell exhaustion.

**Table 2:** Correlation analysis between SIRT7 and relate genes and markers of immune cells in breast cancer.

**Description**	**Gene markers**	**BRCA-Luminal (*****N*** **= 611)**				**BRCA-HER2 (*****N*** **= 67)**				**BRCA-Basal (*****N*** **= 139)**			
		**None**		**Purity**		**None**		**Purity**		**None**		**Purity**	
		**Cor**	***P*-value**	**Cor**	***P*-value**	**Cor**	***P*-value**	**Cor**	***P*-value**	**Cor**	***P*-value**	**Cor**	***P*-value**
B cell	CD19	0.068	9.27e-02	0.105	**1.46e-02**	0.239	5.51e-02	0.182	1.72e-01	0.058	4.94e-01	0.102	2.52e-01
	CD79A	0.049	2.29e-01	0.084	5.09e-02	0.234	5.71e-02	0.189	1.56e-01	0.076	3.74e-01	0.138	1.21e-01
T cell (general)	CD3D	0.031	4.37e-01	0.079	6.46e-02	0.301	1.35e-02	0.3	2.21e-02	0.023	7.84e-01	0.085	3.38e-01
	CD3E	0.014	7.28e-01	0.064	1.38e-01	0.294	1.61e-02	0.284	3.06e-02	−0.010	9.02e-01	0.053	5.52e-01
	CD2	−0.012	7.62e-01	0.03	4.88e-01	0.261	3.33e-02	0.264	4.51e-02	−0.016	8.51e-01	0.047	5.97e-01
CD8+ T cell	CD8A	0.004	9.17e-01	0.045	2.97e-01	0.283	2.08e-02	0.27	3.93e-02	−0.021	8.081-01	0.032	7.19e-01
	CD8B	0.025	5.32e-01	0.069	1.08e-01	0.333	6.17e-03	0.323	1.34e-02	−0.102	2.29e-01	−0.101	2.55e-01
Monocyte	CD86	−0.100	1.34e-02	−0.078	7.05e-02	0.130	2.94e-01	0.132	3.22e-01	0.022	7.97e-01	0.071	4.25e-01
TAM	CCL2	−0.040	3.22e-01	−0.016	7.02e-01	0.174	1.58e-01	0.142	2.87e-01	0.072	3.99e-01	0.098	2.69e-01
	CD68	−0.033	4.18e-01	−0.015	7.19e-01	0.086	4.90e-01	0.094	4.84e-01	0.009	9.20e-01	0.037	6.78e-01
	IL10	−0.060	1.36e-01	−0.04	3.46e-01	0.028	8.23e-01	0.001	9.93e-01	−0.069	4.19e-01	−0.068	4.49e-01
M1 Macrophage	INOS (NOS2)	−0.036	6.71e-01	−0.029	5.06e-01	0.142	2.51e-01	0.201	1.31e-01	−0.036	6.71e-01	−0.069	4.38e-01
	IRF5	0.150	1.81e-04	0.165	**1.13e-04**	0.242	4.91e-02	0.224	9.12e-02	0.117	1.70e-01	0.155	8.09e-02
M2 Macrophage	CD163	−0.069	8.90e-02	−0.043	3.15e-01	−0.053	6.67e-01	−0.096	4.72e-01	−0.095	2.63e-01	−0.111	2.12e-01
	VSIG4	−0.144	3.51e-04	−0.108	**1.16e-02**	−0.161	1.93e-01	−0.15	2.62e-01	−0.063	4.58e-01	−0.075	4.01e-01
	MS4A4A	−0.113	4.83e-03	−0.093	3.04e-02	0.107	3.87e-01	0.111	4.08e-01	−0.047	5.84e-01	−0.041	6.43e-01
Neutrophils	CD66b (CEACAM8)	0.012	7.66e-01	0.025	5.68e-01	−0.010	9.39e-01	−0.076	5.72e-01	−0.080	3.48e-01	−0.12	1.76e-01
	CD11b (ITGAM)	−0.107	7.76e-03	−0.056	1.93e-01	0.072	5.60e-01	0.055	6.83e-01	0.011	8.93e-01	0.023	7.93e-01
	CCR7	0.040	3.16e-01	0.086	5.12e-02	0.205	9.63e-02	0.163	2.26e-01	−0.099	2.43e-01	−0.08	3.68e-01
Natural killer cell	KIR2DL1	0.067	9.48e-02	0.106	**1.31e-02**	0.089	4.74e-01	0.13	3.31e-01	0.094	2.71e-01	0.131	1.42e-01
	KIR2DL3	0.055	1.73e-01	0.056	1.95e-01	0.302	1.31e-02	0.302	2.14e-02	0.083	3.27e-01	0.105	2.38e-01
	KIR3DL1	−0.015	7.03e-01	0.023	5.86e-01	−0.047	7.07e-01	−0.104	4.37e-01	−0.009	9.17e-01	0.024	7.87e-01
	KIR3DL2	0.042	2.95e-01	0.063	1.40e-01	0.181	1.42e-01	0.224	9.14e-02	0.105	2.17e-01	0.181	4.13e-02
	KIR3DL3	0.013	7.47e-01	0.035	4.20e-01	0.111	3.72e-01	0.148	2.69e-01	0.157	6.37e-02	0.203	2.13e-02
	KIR2DS4	0.025	5.36e-01	0.029	5.06e-01	0.277	2.32e-02	0.284	3.10e-02	0.147	8.29e-02	0.244	5.49e-03
Dendritic cell	HLA-DPB1	−0.015	7.10e-01	0.046	2.80e-01	0.286	1.91e-02	0.303	2.06e-02	0.040	6.39e-01	0.092	3.01e-01
	HLA-DQB1	−0.003	9.32e-01	0.046	2.80e-01	0.286	2.84e-02	0.276	3.61e-02	0.124	1.45e-01	0.168	5.73e-02
	HLA-DRA	−0.106	8.56e-03	−0.063	1.42e-01	0.164	1.84e-01	0.165	2.15e-01	0.011	8.97e-01	0.05	5.77e-01
	HLA-DPA1	−0.105	8.89e-03	−0.068	1.13e-01	0.107	3.87e-01	0.102	4.46e-01	−0.022	7.92e-01	0.016	8.55e-01
	BDCA-1(CD1C)	−0.131	1.11e-03	−0.114	**7.69e-03**	0.207	9.27e-02	0.202	1.29e-01	−0.097	2.56e-01	−0.099	2.67e-01
	CD11c (ITGAX)	−0.015	7.14e-01	0.02	6.47e-01	0.248	4.33e-02	0.207	1.18e-01	0.045	5.95e-01	0.093	2.97e-01
Th1	T-bet (TBX21)	0.051	2.05e-01	0.103	**1.00e-02**	0.233	5.82e-02	0.214	1.07e-01	−0.019	8.24e-01	0.018	8.42e-01
	STAT4	−0.088	2.92e-02	−0.054	2.10e-01	0.324	7.80e-03	0.315	1.61e-02	−0.087	3.05e-01	−0.075	3.97e-01
	STAT1	−0.050	2.13e-01	−0.02	6.40e-01	0.117	3.46e-01	0.122	3.61e-01	0.028	7.41e-01	0.082	3.55e-01
	IFN-γ (IFNG)	0.009	8.19e-01	0.03	4.83e-01	0.222	7.13e-02	0.23	7.83e-02	0.040	6.40e-01	0.092	3.03e-01
	TNF-α (TNF)	0.001	9.84e-01	0.007	8.79e-01	−0.004	9.73e-01	−0.117	3.81e-01	0.070	4.12e-01	0.07	4.30e-01
Th2	GATA3	0.061	1.33e-01	0.039	3.62e-01	0.102	4.12e-01	0.2	1.31e-01	0.079	3.52e-01	0.117	1.87e-01
	STAT6	0.019	6.40e-01	0.02	6.47e-01	−0.081	5.14e-01	−0.011	9.36e-01	0.019	8.22e-01	0.045	6.17e-01
	STAT5A	−0.061	1.31e-01	−0.025	5.60e-01	0.212	8.56e-02	0.242	6.66e-02	0.117	1.69e-01	0.135	1.29e-01
	IL13	0.012	7.67e-01	0.026	5.47e-01	0.247	4.36e-02	0.287	2.92e-02	−0.064	4.52e-01	−0.042	6.38e-01
Tfh	BCL6	−0.054	1.79e-01	−0.053	2.16e-01	−0.030	8.09e-01	−0.066	6.25e-01	0.023	7.86e-01	0.042	6.38e-01
	IL21	0.013	7.51e-01	0.037	3.94e-01	0.093	4.53e-01	0.084	5.29e-01	−0.047	5.83e-01	−0.023	7.99e-01
Th17	IL17A	−0.045	2.67e-01	−0.036	3.98e-01	0.106	3.94e-01	0.158	2.37e-01	−0.031	7.15e-01	−0.001	9.90e-01
Treg	FOXP3	0.078	5.40e-02	0.12	**5.06e-03**	0.275	2.47e-02	0.25	5.81e-02	0.026	7.64e-01	0.088	3.24e-01
	CCR8	−0.058	1.52e-01	−0.036	4.05e-01	0.156	2.06e-01	0.172	1.98e-01	−0.118	1.65e-01	−0.105	2.39e-01
	STAT5B	−0.049	2.21e-01	−0.036	4.00e-01	0.060	6.28e-01	0.022	8.69e-01	−0.047	5.78e-01	−0.048	5.93e-01
	TGFβ (TGFB1)	0.033	4.19e-01	0.074	8.29e-02	0.121	3.28e-01	0.181	1.73e-01	−0.062	4.64e-01	−0.061	4.95e-01
T cell exhaustion	PD-1 (PDCD1)	0.088	2.95e-02	0.134	**1.74e-03**	0.328	6.98e-03	0.318	1.51e-02	0.096	2.57e-01	0.169	5.70e-02
	CTLA4	0.025	5.40e-01	0.052	2.29e-01	0.261	3.34e-02	0.234	7.71e-02	−0.003	9.68e-01	0.05	5.72e-01
	TIM-3 (HAVCR2)	−0.103	1.06e-02	−0.075	7.84e-02	0.134	2.81e-01	0.168	2.07e-01	0.005	9.56e-01	0.05	5.77e-01
	GZMB	0.068	9.16e-02	0.108	**1.14e-02**	0.268	2.87e-02	0.262	4.69e-02	0.052	5.43e-01	0.098	2.69e-01

*Th, T helper cell; Tfh, Follicular helper T cell; Treg, regulatory T cell; Cor, R value of Spearman's correlation; None, correlation without adjustment. Purity, correlation adjusted by purity. Bold values indicate that P < 0.05 when Cor > 0.1*.

### Correlation Analysis Between Sirtuin 7 Expression and Markers of Different Subsets of Immune Cells

**Figure 5:**
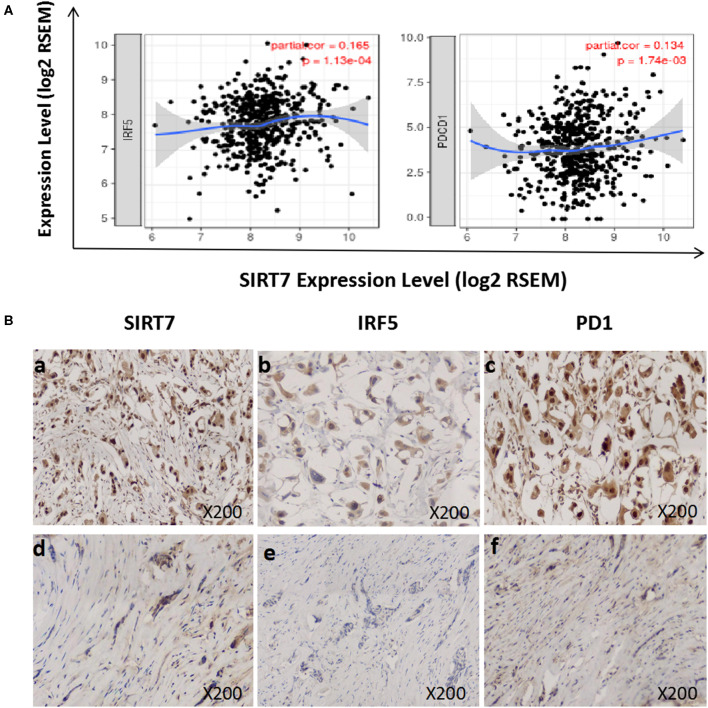
Correlation between sirtuin (SIRT)7 expression and immune infiltration levels of M1 macrophages and T cell exhaustion in breast cancer-luminal. **(A)** SIRT7 expression is significantly positively correlated with M1 macrophages (*r* = 0.165, *p* = 1.13e-04) and T cell exhaustion (*r* = 0.134, *p* = 1.74e-03). **(B)** Tumor infiltration of M1 macrophages and T cell exhaustion in breast cancer-luminal. The expression of SIRT7 (a: + + +, d: +). The expression of IRF5 (b: ++, e: –). The expression of PD1 (PDCD1) (c: + + +, f: +). The expression density of SIRT7, PD1, and IRF5 in breast cancer tissue was quantitated by scoring staining intensity, including negative (–) and weak (+) staining, moderate (++) and strong (+ + +) staining, respectively.

We investigated the relationship between SIRT7 expression and tumor-infiltrating of immune cells (M1 macrophages and T cell exhaustion) based on the expression level of immune marker genes in the TIMER databases (Figure 5A). We found that the expression of SIRT7 was positively correlated with the expression of IRF5 (M1 macrophages marker) and PD1 (PDCD1) (T cell exhaustion marker) in breast cancer-luminal. These results suggested that the expression of SIRT7 was related to M1 macrophages and T cell exhaustion infiltration.

Next, we further analyzed the correlation between SIRT7 expression and these markers by immunohistochemistry, and tumor infiltration of M1 macrophages and T cell exhaustion were shown in Figure 5B. The levels of the expression were quantitated by scoring staining intensity, including negative (–) and weak (+) staining, moderate (++) and strong (+ + +) staining, respectively. We found that SIRT7 mainly localized in the nucleolus and nucleoplasm. SIRT7 showed strong expression in most breast cancer-luminal subtype samples, and few samples showed moderate expression. Interestingly, the expression levels of IRF5 and PD1 were high in SIRT7 strong expression samples, while the expression levels of IRF5 and PD1 were relatively low in SIRT7 weak expression samples. The results further revealed that IRF5 and PD1 tend to express in a positive correlation way, suggesting that the high expression of SIRT7 relates to high infiltration levels of M1 macrophages and T cell exhaustion.

## Discussion

In this study, an extensive bioinformatics examination was used to comprehensively analyze the SIRT7 expression pattern, investigate its association with breast cancer prognosis, and predict the correlation between SIRT7 expression and the immune infiltration levels of different immune cells in breast cancer-luminal.

SIRT7, a member of the SIRT family of NAD^+^-dependent protein deacetylases, is a key mediator of many cellular activities (37). The SIRT family is associated with malignant progression of tumors, and SIRT7 is supposed to be involved in lymph node metastasis of breast cancer (38). The expression of SIRT7 is related to cell growth. It is highly expressed in metabolically active cells, while it is lowly expressed or not expressed in non-proliferative cells (20, 39, 40). Some studies suggested the carcinogenic potential of SIRT7, whose expression levels were associated with several cancers, including breast, ovarian, and lung cancers, thereby classifying SIRT7 as a carcinogenic gene (41–44). SIRT7 is also a potential prognostic factor for breast cancer (45). However, the potential functions of SIRT7 in tumor progression and tumor immunology remain unclear.

We examined the expression levels of SIRT7 in breast cancer using TIMER, GEPIA, and HPA databases. We found that SIRT7 was highly expressed in breast cancer, which is consistent with Geng et al. (46). Some studies reported that age at diagnosis was an independent prognostic factor for breast cancer (47, 48), especially for breast cancer-luminal A. However, the correlation between age and SIRT7 expression remains unclear (49). In multivariate analysis, after adjusting for age, nodal status, ER, PR, HER2, and triple-negative status, we found that SIRT7 expression was significantly associated with age, nodal status, ER, PR, and triple-negative status. These results suggest that SIRT7 expression may be a potential diagnostic indicator of breast cancer. Different molecular types of breast cancer are associated with different prognostic survival of patients (50). Although SIRT7 expression is associated with poor prognosis in some cancers, such as colorectal cancer (51), the effect of SIRT7 expression on the prognosis of breast cancer subtypes is unclear. To definitively address this question, we classified breast cancer into three main subtypes, refer to the study of Polyak and Filho. (3). We believe that this classification is practical, straightforward, informative, and clinically useful, exhibiting considerable differences between subtypes. To determine whether SIRT7 can be used as a prognostic biomarker, we analyzed the expression of SIRT7 in different subtypes from the Kaplan-Meier plotter. There were significant differences in clinical and pathological features and prognosis between different subtypes. Notably, we found that the high expression of SIRT7 in breast cancer-luminal A was associated with poor prognosis, suggesting that the expression of SIRT7 will affect the prognosis of breast cancer.

Another important aspect of this study is that SIRT7 expression is associated with the immune infiltration levels in breast cancer-luminal. Several studies reported that TILs were currently considered to be biomarkers highly associated with breast cancer. Generally, it is recognized that the infiltration of TILs was related to the prognosis in breast cancer, and that adjuvant treatment is relatively effective (52, 53). In addition, several clinical studies evaluated the predictive importance of TILs for prognosis in breast cancer (54). However, the relationship between SIRT7 and immune infiltration in breast cancer-luminal remains unclear. Therefore, in this study, we comprehensively analyzed SIRT7 expression and associated immune infiltration and investigated the relationship between SIRT7 expression and immune infiltration levels in breast cancer-luminal. To the best of our knowledge, this study is the first to evaluate the association between SIRT7 expression and the levels of immune infiltration in breast cancer subtypes, especially in breast cancer-luminal. Our results demonstrated a positive correlation between SIRT7 expression and the levels of M1 macrophages (marker: IRF5) and T cell exhaustion (marker: PD1) infiltration. Macrophages are a group of differentiated immune cells and classify as M1 macrophages and M2 macrophages (55). They play an important role in development, homeostasis, and immunity (56). IRF5 is a marker of M1 macrophages and plays the role of regulating tumor infiltration (57). Previous studies showed that IRF5 changed the immune microenvironment of tumors by regulating the expression of pro-inflammatory and anti-inflammatory cytokines/chemokines in breast cancer (58, 59). Our studies observed that an increase in SIRT7 expression was correlated with the M1 macrophage marker IRT5. It suggests that there is a specific correlation between SIRT7 expression and immune infiltrating of M1 macrophages. Furthermore, SIRT7 expression was significantly correlated with T cell exhaustion markers such as PD-1. It is known that PD1/PDL1 are important immune checkpoint components, which mainly regulate the function of tumor cells and TILs (60), and the PD1/PDL1 axis was proved to be a promising therapeutic target in aggressive breast cancers (61). Moreover, Woo et al. (62) reported that PD-1 might play an important role in the tolerance of tumor antigens. These correlations may indicate the potential mechanism of SIRT7 regulating T cell functions. Taken together, these findings suggest that SIRT7 expression is related to the immune infiltration levels in breast cancer, especially breast cancer-luminal, providing a direction for further research.

A limitation is that our data do not include detailed information of body mass index, comorbidities, and treatment options. The data we collected in this study lack this sort of information, but we promise we will pay more attention to the integrity of patient baseline information in the following studies, and we will further confirm the estimated results in experiments and study the mechanism of this gene in breast tumor immunity.

In summary, the high expression of SIRT7 indicates poor prognosis in breast cancer-luminal, as well as increased immune infiltration levels of M1 macrophages and T cell exhaustion, suggesting that SIRT7 may serve as a prognostic biomarker associated with immune infiltration in breast cancer-luminal.

## Data Availability Statement

Publicly available datasets were analyzed in this study. This data can be found here: https://cistrome.shinyapps.io/timer/, https://www.Proteinatlas.org/, http://ualcan.path.uab.edu/, http://bcgenex.centregauducheau.fr/, http://kmplot.com/analysis/, http://gepia.cancer-pku.cn/.

## Author Contributions

QH and ZL conceived the project, participated in data analysis and wrote the manuscript, they contributed equally to this work. LC and FY participated in discussion and language editing. LC and NX reviewed the manuscript. LC supervised this project.

## Conflict of Interest

The authors declare that the research was conducted in the absence of any commercial or financial relationships that could be construed as a potential conflict of interest.
